# Ectopic bronchogenic cyst of the gastric cardia considered to be a gastrointestinal stromal tumor before surgery: a case report

**DOI:** 10.1186/s12893-020-00704-z

**Published:** 2020-03-02

**Authors:** Jianchun Xiao, Ruopeng Zhang, Wanqi Chen, Bin Wu

**Affiliations:** 1 Peking Union Medical College Hospital, Beijing, 100730 China; 2 Peking Union Medical College, Beijing, China

**Keywords:** Bronchogenic cyst, Preoperative diagnosis, Gastrointestinal stromal tumor, Wedge resection

## Abstract

**Background:**

We herein report a rare case of an ectopic bronchogenic cyst of the gastric cardia. The initial diagnosis was a gastrointestinal stromal tumor (GIST); however, postoperative pathologic examination confirmed that it was a bronchogenic cyst.

**Case presentation:**

A 62-year-old woman visited our hospital for abdominal pain. The diagnosis prior to surgery was a GIST. Computed tomography imaging showed that the mass was located in the gastric cardia on the side of the lesser curvature. During the surgical exploration, it was noted that the tactility of the mass was not consistent with a GIST. Thus, we decided to perform local resection of the mass and part of the gastric wall without wedge resection. The pathological examination revealed a bronchogenic cyst.

**Conclusions:**

This case suggests that a bronchogenic cyst should be considered as a differential diagnosis of a GIST. It is also a unusual but necessary situation should be considered when explaining the etiology of a bronchogenic cyst.

## Background

A bronchogenic cyst is a cystic mass caused by congenital dysplasia of the respiratory system. These cysts can be divided into three types according to the location of the disease: intrapulmonary, mediastinal, and ectopic [1]. The ectopic type is rare. It can occur in the neck, brain, dural membrane, and abdominal cavity [2]. The etiology of bronchogenic cysts remains unclear. Ectopic bronchogenic cysts show no specific signs on computed tomography (CT) or magnetic resonance imaging (MRI). Thus, the misdiagnosis rate of this disease may reach 40 to 60%, and the final diagnosis depends on the pathological examination.

## Case presentation

**Fig. 1:**
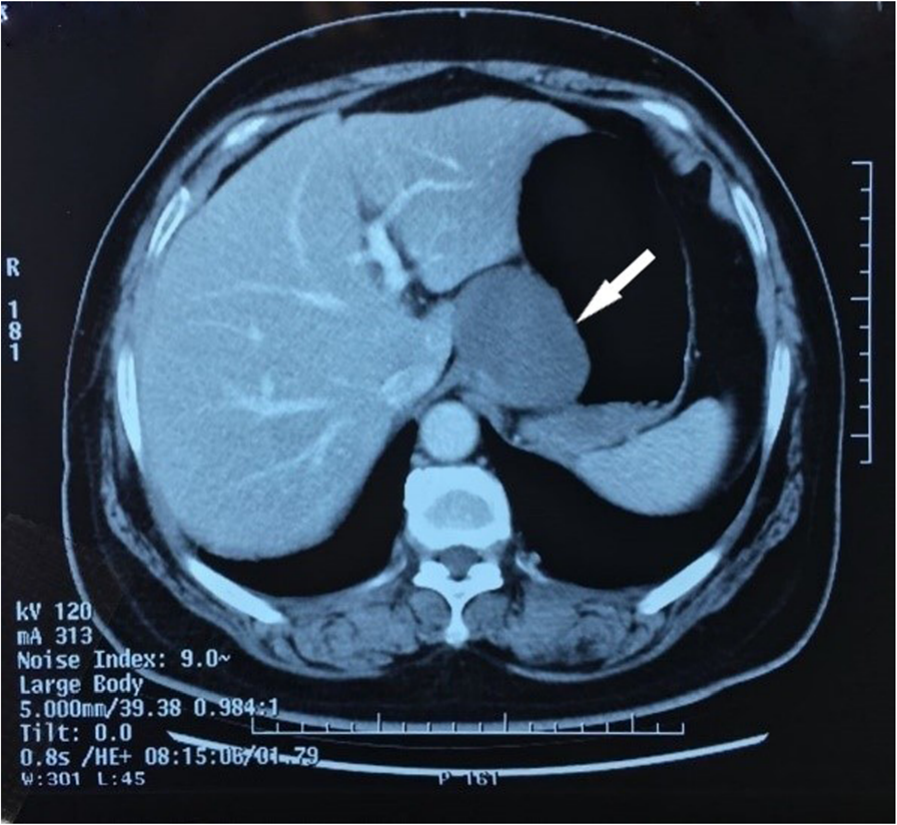
Abdominal enhanced computed tomography. The mass appeared as a quasi-circular object adjacent to the lesser curvature. Its size was approximately 6.4 × 4.9 cm, and it had a uniform density with no obvious enhancement

**Fig. 2:**
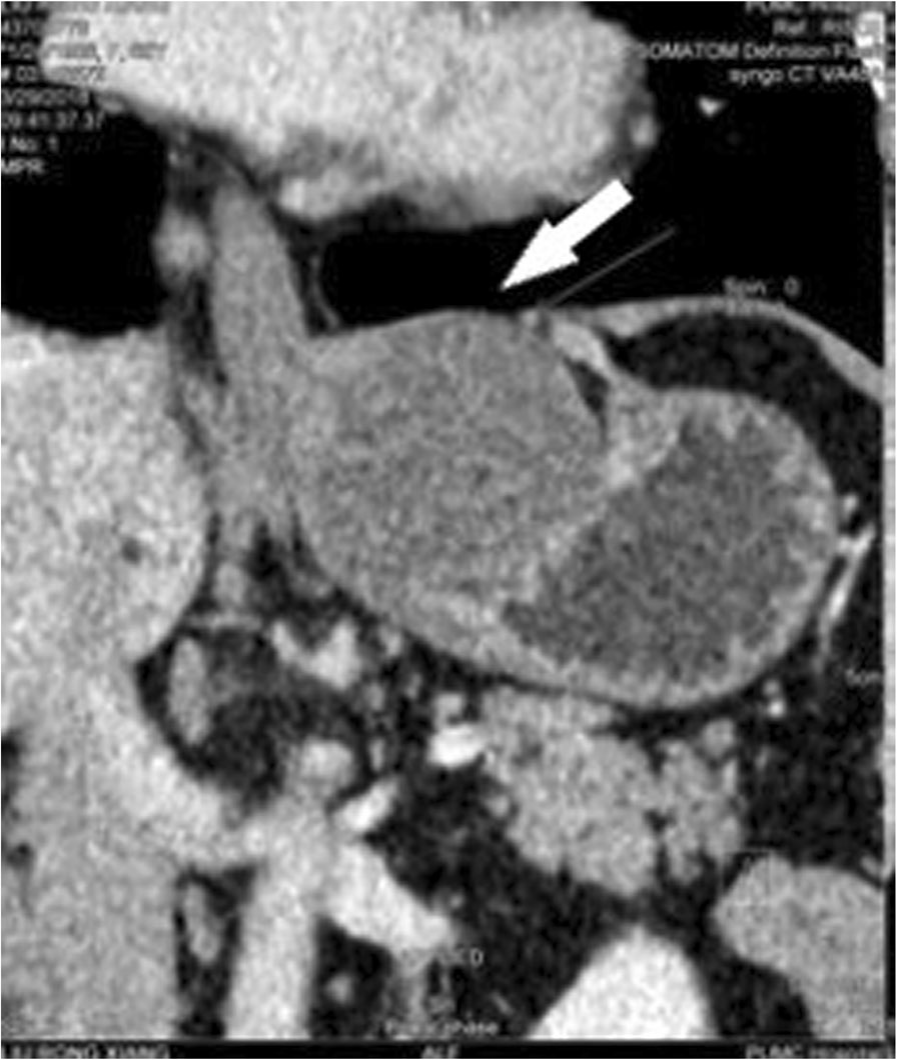
Gastric reconstruction imaging. A gastric filling well with a low-density shadow was observed on the side of the lesser curvature close to the gastric cardia, and nodular calcification was present along the edge

A 62-year-old woman presented to our hospital with a 1-year history of persistent pain in the right lower abdomen. No abnormalities were observed during the physical examination, and no palpable abdominal mass was identified. Abdominal enhanced CT (Fig. 1) and gastric reconstruction imaging (Fig. 2) revealed a gastric filling well, a low-density shadow on the lesser curvature of the gastric cardia with nodular calcifications on the edge, and a mass of approximately 6.4 × 4.9 cm with uniform density. No obvious enhancement, thickening of the gastric wall, or strengthening of the gastric mucosa was observed. No abnormalities were found by electronic gastroscopy. We considered the mass to be a GIST, although we did not exclude the possibility of other diagnoses. Exploratory surgery was performed for diagnosis and treatment.

**Fig. 3:**
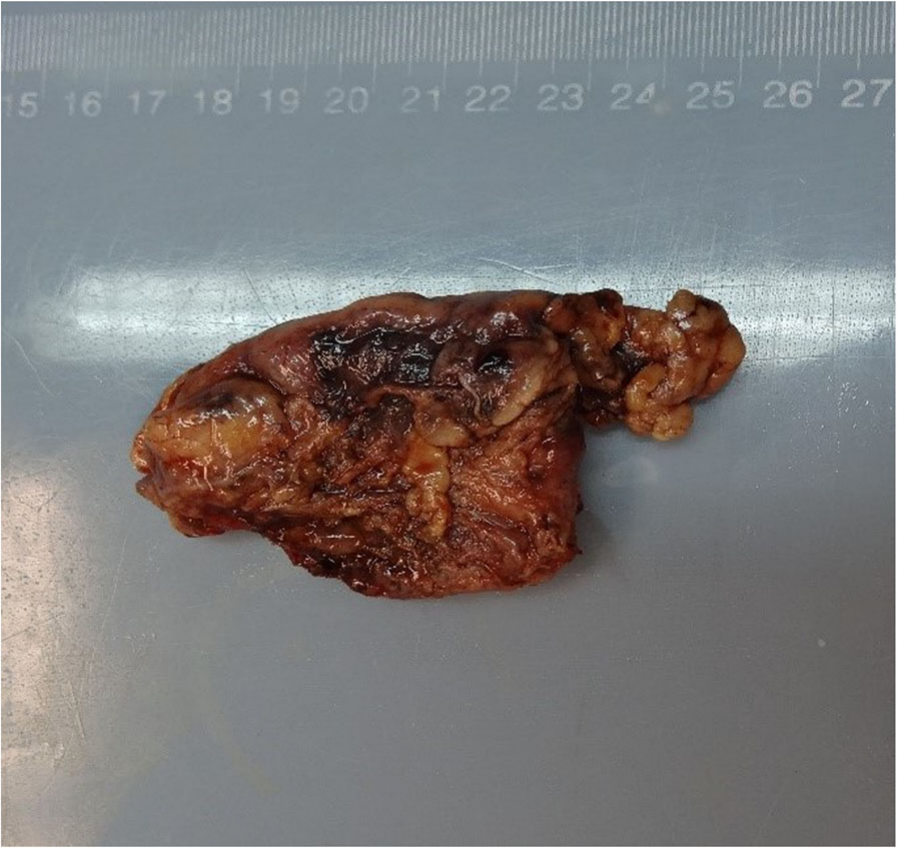
Appearance of the mass specimen

**Fig. 4:**
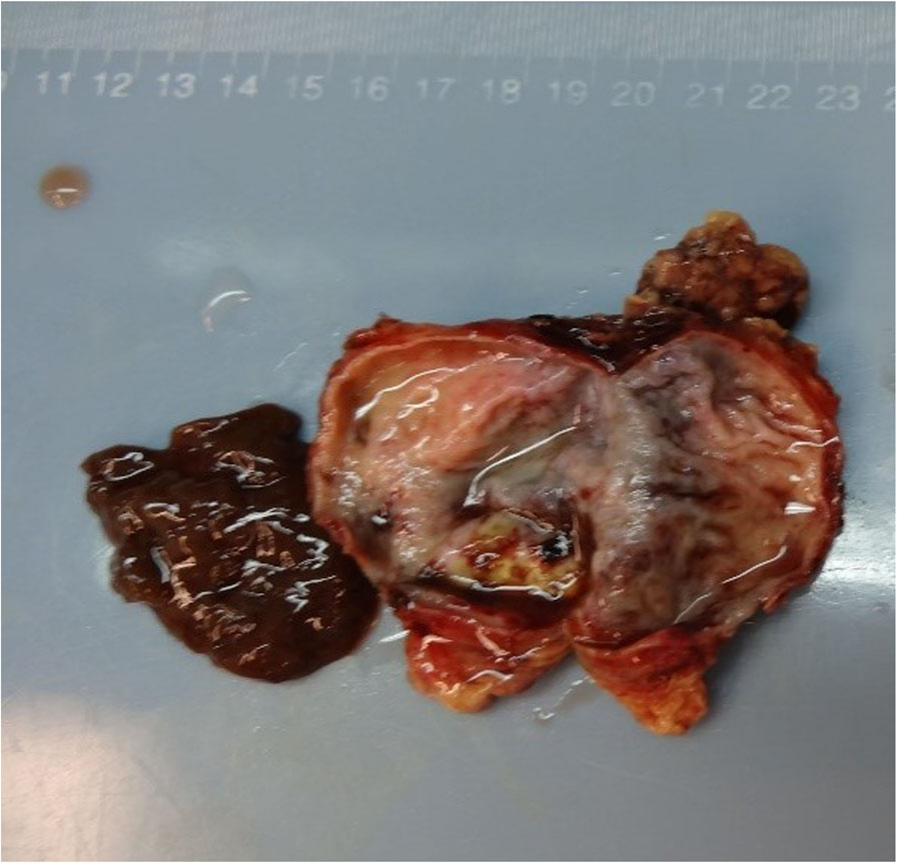
Cut surface of the mass. The mass substance mainly consisted of cystic components

The patient agreed to undergo laparoscopic surgery. After careful exploration, we found that the mass was located on the side of the lesser curvature, near the cardia. It was prominent on the stomach wall, and its diameter was approximately 6 cm. The mass was soft and mainly consisted of cystic components, which did not conform to the characteristics of a GIST. Therefore, we exposed the visual field of the cardia and endoscopically resected the muscular layer and mass. The mass was not significantly adhered to the surrounding tissues, and the separation was smooth; neither removal of the surrounding tissues nor lymph node dissection was needed. The specimen (Figs. 3 and 4) was sent out for pathological examination. We performed seromuscular suture of the gastric wall after the resection. The patient recovered well and her cardiac function was normal after operation. She was discharged 6 days postoperatively. A follow-up consultation 3 months after the operation indicated that the patient was in good condition and had no abdominal pain or other complaints or complications.

**Fig. 5:**
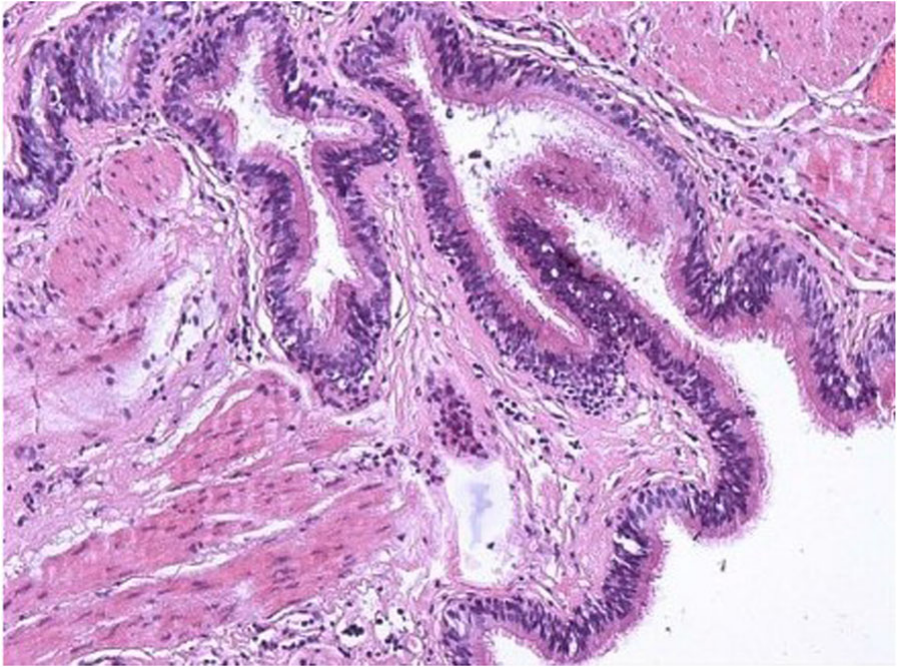
Pathological section of the mass. The mass had a cystic surface, and dark brown, soft, brittle contents were present under the capsule. The pathological diagnosis was a bronchogenic cyst

The postoperative pathological examination findings (Fig. 5) revealed that the specimen was a single nodule of 7.0 × 4.5 × 1.5 cm and had a cystic surface. The contents under the capsule were dark brown, soft, and brittle; the capsule wall was smooth and 0.1 to 0.2 cm thick. A partially mucosal, slightly gray area of 2 × 1 cm was also present The pathological results indicated that the mass was a bronchogenic cyst. The patient underwent regular postoperative follow-up in our hospital for 1 year, during which time she experienced no discomfort or recurrence of the tumor on CT.

## Discussion and conclusion

GISTs are the most common type of mesenchymal tumor found in the gastrointestinal tract. They are usually found in the stomach and proximal small intestine but can occur in any part of the digestive tract [3] [4]. Wedge resection of the stomach wall is the most commonly performed procedure for the treatment of gastric GISTs, but there is a risk of cardiac dysfunction when wedge resection is performed near the cardia. For benign masses, local excision should be considered rather than wedge resection [5].

In this case, we diagnosed the mass as a GIST before surgery, but intraoperative examination revealed that it did not have the typical characteristics of a GIST. Because of the risk of peritoneal dissemination by percutaneous biopsy [6], surgical exploration was the best option in this case. GISTs are brittle and bleed easily, but the operation in our case revealed that the mass mainly consisted of cystic components. Therefore, we concluded that the mass was a cyst rather than a GIST and performed local excision.

The pathological examination of this specimen revealed that it was a bronchogenic cyst. CT and MRI can help us determine the nature of the mass, but in the end we need intraoperative exploration and pathological results to confirm the diagnosis. For suspected GISTs, MRI or endoscopic ultrasound-guided fine needle aspiration biopsy can help to further identify the origin of the tumor. The final diagnosis depends on the pathological examination [7, 8]. According to existing theories, a bronchogenic cyst develops when tissue cells fall off or travel around the neck during embryonic development, and these cells remain relatively close to their original position. Therefore, bronchogenic cysts near the cardia are very rare, and their existence reminds us that some mechanisms of bronchogenic cyst formation have not been discovered. The most effective treatment for bronchogenic cysts is surgical resection, and early surgical treatment is ideal especially for patients with symptoms caused by tumor compression. The surgeon should strive to avoid damaging the capsule wall and to ensure complete resection to reduce the recurrence rate [9]. If bronchogenic cysts are not completely excised, they may recur. Therefore, postoperative follow-up is generally required to determine whether the patient’s symptoms and imaging findings indicate tumor recurrence.

In conclusion, we have herein described a rare case of an ectopic bronchogenic cyst of the gastric cardia. The initial diagnosis was a GIST, but postoperative pathological examination confirmed that it was a bronchogenic cyst. This case suggests that a bronchogenic cyst should be considered as a differential diagnosis of a GIST. It is also a unusual but necessary situation should be considered when explaining the etiology of a bronchogenic cyst.

## Data Availability

All data are contained within the manuscript file.

## Abbreviations

CT: Computed tomography
GIST: Gastrointestinal stromal tumor
MRI: Magnetic resonance imaging
